# Supplementary material for: Identification of photoperiod-induced specific miRNAs in the adrenal glands of Sunite sheep (Ovis aries)
Source: Front Vet Sci. 2022 Jul 22;9:888207. doi: 10.3389/fvets.2022.888207 (PMC9354845; doi:10.3389/fvets.2022.888207)
Supplement: Supplementary file 1 [file Data_Sheet_1.ZIP › Supplementary file/miRDeep2 file.zip!/miRDeep2 file/Novel_Structure/LP42A1/Novel_655.pdf]

Provisional ID : NC_019480.2_31781
Score total : 0.5
Score for star read(s) : -1.3
Score for read counts : 0
Score for mfe : 0.2
Score for randfold : 1.6
Score for cons. seed :
Total read count : 20
Mature read count : 20
Loop read count : 0
Star read count : 0

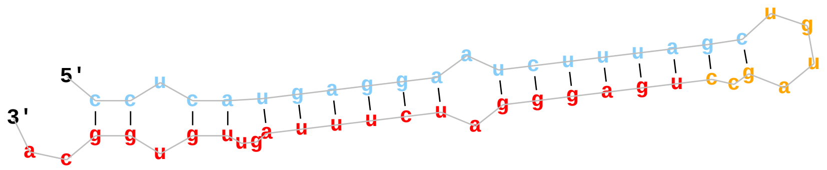

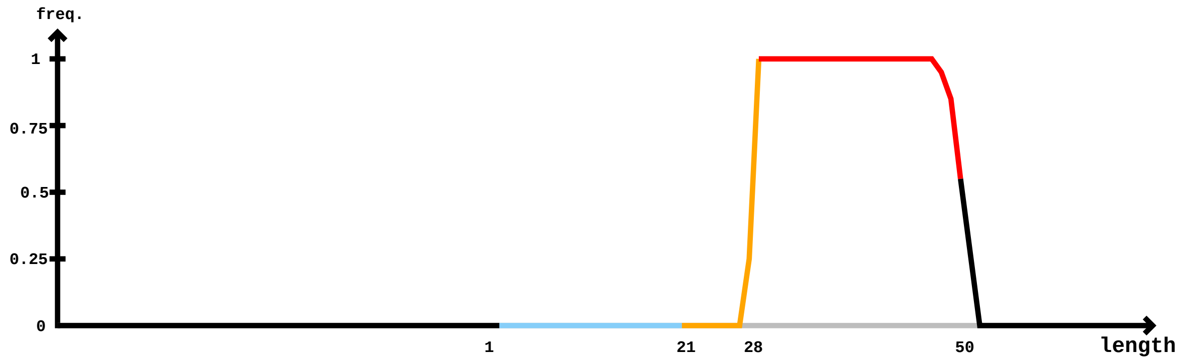


```
                                              Star                 Mature
5'- uaaauauucuaauuuauuuauuugacuguguugggucuuaguugccucaugaggaaucuuuagcuguagccugagggaucuuuaguuguggcaugugggaucuaguucccug   -3'    exp
    ((((((........))))))..........((((((((((..((((.((((((((.((((((((....).))))))).))))))..)).))))..)))))))))).......    reads      mm        sample
    ......................................................................Augagggaucuuuaguugugg.....................    2          1         seq
    ......................................................................cugagggaucuuuaguuguggc....................    2          0         seq
    ......................................................................Augagggaucuuuaguuguggca...................    1          1         seq
    .......................................................................ugGgggaucuuuaguugug......................    1          1         seq
    .......................................................................ugUgggaucuuuaguuguggc....................    4          1         seq
    .......................................................................ugGgggaucuuuaguuguggca...................    3          1         seq
    .......................................................................ugUgggaucuuuaguuguggca...................    7          1         seq
```
